# Comparison of semi-quantitative and quantitative methods for diagnosis of catheter-related blood stream infections: a systematic review and meta-analysis of diagnostic accuracy studies

**DOI:** 10.1017/S0950268820001673

**Published:** 2020-07-27

**Authors:** Yan Zhang, Li Yang, Yanmei Chu, Linlin Wu

**Affiliations:** 1 Department of ICU, Zaozhuang Municipal Hospital, Zaozhuang 277100, Shandong Province, P.R. China; 2 Postpartum Health Care Pelvic Floor Function Diagnosis and Treatment Center, Zaozhuang Maternity and Child Health Care Hospital, Zaozhuang 277100, Shandong Province, P.R. China; 3 Operating Room, Zaozhuang Maternity and Child Health Care Hospital, Zaozhuang 277100, Shandong Province, P.R. China

**Keywords:** Catheter-related blood stream infections, meta-analysis, quantitative culture, semi-quantitative culture, validation studies

## Abstract

Catheter-related blood-stream infections (CRBSIs) are the most common healthcare-associated blood-stream infections. They can be diagnosed by either semi-quantitative or quantitative methods, which may differ in diagnostic accuracy. A meta-analysis was undertaken to compare the diagnostic accuracy of semi-quantitative and quantitative methods for CRBSI. A systematic search of Medline, Scopus, Cochrane and Embase databases up to January 2020 was performed and subjected to a QUADAS (quality assessment of diagnostic accuracy studies 2) tool to evaluate the risk of bias among studies. The pooled sensitivity and specificity of the methods were determined and heterogeneity was evaluated using the *χ*^2^ test and *I*^2^. Publication bias was assessed using a Funnel plot and the Egger's test. In total, 45 studies were analysed with data from 11 232 patients. The pooled sensitivity and specificity of semi-quantitative methods were 85% (95% CI 79–90%) and 84% (95% CI 79–88%), respectively; and for quantitative methods were 85% (95% CI 79–90%) and 95% (95% CI 91–97%). Considerable heterogeneity was statistically evident (*P* < 0.001) by both methods with a correspondingly symmetrical Funnel plot that was confirmed by a non-significant Deek's test. We conclude that both semi-quantitative and quantitative methods are highly useful for screening for CRBSI in patients and display high sensitivity and specificity. Quantitative methods, particularly paired quantitative cultures, had the highest sensitivity and specificity and can be used to identify CRBSI cases with a high degree of certainty.

## Introduction

Central venous or peripheral artery catheters are used for managing critically-ill or emergency patients admitted to intensive care units (ICUs) or emergency units of tertiary care centres. The most common complication associated with their use is catheter-related blood-stream infection (CRBSI) [1, 2], which constitute a major part of healthcare-associated bacteraemia and are associated with significant morbidity, mortality and excess economic burden to the hospitals [3, 4]. Hence, preventing such infections by appropriate implementation of diagnostic and therapeutic practices is highly important.

CRBSI diagnoses remain challenging as the most common signs of these infections are often non-specific-like fever and chills; inflammation at the catheter-insertion site has a sensitivity of only 8% [5]. Microbiological evidence to establish the central venous or peripheral arterial catheters as the source of bacteraemia is therefore required for diagnosis confirmation; however, despite intensive research, gold-standard methods are lacking. A semi-quantitative diagnostic technique was first recommended in 1977 by Maki *et al*. [6] in which, a catheter tip segment was cultured on the surface of a blood agar plate to detect bacterial contamination. This became the benchmark method and is widely used as a reference standard against which other diagnostic methods for CRBSIs are compared. However, false-negative results may be obtained with this technique for some patients with endoluminal colonisation as it detects only microorganisms present on the surface or outer layer of external catheters. Newer quantitative techniques overcome these limitations and are able to detect both exo- and endo-luminal organisms [7]. The diagnostic accuracy of both techniques has been examined in tertiary care settings with widely varying results, however systematic studies of their comparative diagnostic accuracy are lacking and the most recent evaluation dates back to 2005 [8]. The meta-analysis reported here was therefore designed to provide an updated evaluation of the diagnostic accuracy of semi-quantitative and quantitative methods for CRBSIs.

## Methods

### Types of studies and participants

We selected all studies examining the diagnostic accuracy of either semi-quantitative or quantitative methods for CRBSIs using catheter segments or blood culture, irrespective of study design. All studies included reported sensitivity and specificity values or provided data to allow calculation. They comprised published full-text articles, short communications and conference abstracts. Unpublished studies, thesis reports and those with sample sizes of <10 subjects were omitted. Participants were patients suspected of having a CRBSI in medical, surgical or neonatal ICUs of a tertiary care hospital, irrespective of their age groups or comorbid status. The reference standard was the isolation of the same microbial species from catheters and blood cultures.

### Outcome measures

Pooled sensitivity, specificity, diagnostic odds ratio (DOR), likelihood ratio positive (LRP) and likelihood ratio negative (LRN) from the studies were calculated.

### Search strategy

We performed a systematic and extensive electronic search from inception to January 2020 in databases and search engines (Medline, Embase, Scopus, Cochrane library, Google Scholar and ScienceDirect) without language restriction. Medical subject headings (MeSH) were applied along with free-text search terms (e.g. ‘Validation Studies’, ‘Blood Stream Infections’, ‘Intravascular Catheterization’, ‘Catheter Associated Blood Stream Infections’, ‘Nosocomial Infections’, ‘Quantitative Methods’, ‘Semi-Quantitative Methods’ ‘Sensitivity’, ‘Specificity’, ‘Diagnosis’, ‘Roll Plate Method’, ‘Sonication’ and ‘Diagnostic Accuracy Studies’); the resulting relevant articles were included in the review.

### Selection of studies

Two authors independently performed the primary screening of title, keywords and abstracts, and retrieved full-text articles for the relevant studies. They then undertook a secondary screening of the articles to select those satisfying the inclusion criteria. Conflicts of opinion were resolved either by consultation with a third author or through consensus.

### Data extraction and management

The primary investigator extracted the data from the selected studies pertaining to the study setting, design, participants, inclusion and exclusion criteria reference standards, index test, total participant numbers, and criteria for positivity, sensitivity and specificity. Finally, we compared the data in the review and the study reports to ensure correct entries.

### Risk of bias assessment in included studies

The quality assessment of diagnostic accuracy studies 2 (QUADAS 2) tool was used to appraise the risk of bias among studies [9]. This tool consists of the following domains: patient selection bias, conduct and interpretation of index test and reference standard, and time interval of outcome assessments. Studies were graded as low, high or unclear based on the presence of any bias.

### Statistical analysis

The meta-analysis was carried out using the STATA 14.2 software (StrataCorp, CollegeStation, TX, USA). We calculated pooled estimates of sensitivity, specificity, LRN, LRP and DOR for the semi-quantitative and quantitative methods using the bivariate method. A summary receiver operator characteristic curve (sROC) was constructed to determine the area under the curve (AUC); an AUC value closer to 1 being indicative of a better diagnostic value. Graphical representations were plotted of sensitivity and specificity of individual study-specific and pooled estimates using a forest plot. We determined the clinical values of the semi-quantitative and quantitative methods based on a Likelihood Ratio (LR) scattergram, and the probability of patients having CRBSI using Fagan plots. Heterogeneity was represented graphically using a bivariate boxplot and tested using *χ*^2^ and *I*^2^ statistics. The source of heterogeneity was explored after subgroup analyses using study-related covariates such as type of diagnostic test, country and region. Publication bias was assessed using Deek's test and graphically depicted in a funnel plot. Some statistical analyses were performed using the Midas Command package.

## Results

### Selection of studies

**Fig. 1.**
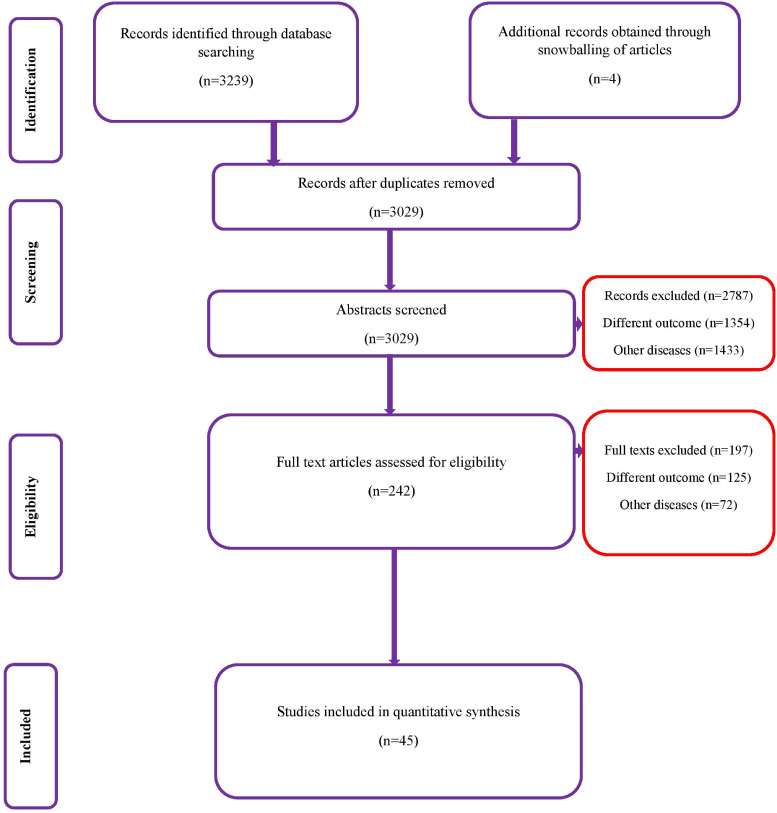
PRISMA flowchart.

A total of 3239 records (1264 studies from Medline, 947 from Scopus, 804 from Embase and 224 from the Cochrane library) of studies were identified on the diagnostic accuracy of semi-quantitative and quantitative methods for CRBSI diagnosis (from inception till January 2020). After the first screening stage, 242 relevant studies were retrieved and full-text of these articles was assessed against the eligibility criteria. Finally, 45 studies with 11 232 participants that met the inclusion criteria were included (Fig. 1) [6, 7, 10–52].

### Characteristics of the selected studies

Supplementary Table S1 shows the characteristics of the studies in our analyses. Most studies (39 out of 45) [11–26, 28–30, 33–43, 45–52] were prospective in nature, 26 studies [7, 10, 12, 16, 18–20, 24, 28–31, 34–36, 38, 40, 42, 43, 46–48, 50, 52] reported on the diagnostic accuracy of the semi-quantitative method and 30 [7, 11–15, 17, 21–23, 25–27, 29, 32–34, 37–47, 50, 51] on quantitative methods such as catheter segment, IVD-drawn or paired lysis cultures. Three studies were conducted in Australia, and the rest were from the Americas (USA and Brazil), France, Spain and the UK. Sample sizes of participants varied from 12 to 1000 (5699 in studies reporting diagnostic accuracy of semi-quantitative methods and 5533 reporting diagnostic accuracy of quantitative methods). Most studies used qualitative catheter segment and qualitative paired blood cultures as reference standards for the final diagnosis.

### Methodological quality of the studies

**Fig. 2.**
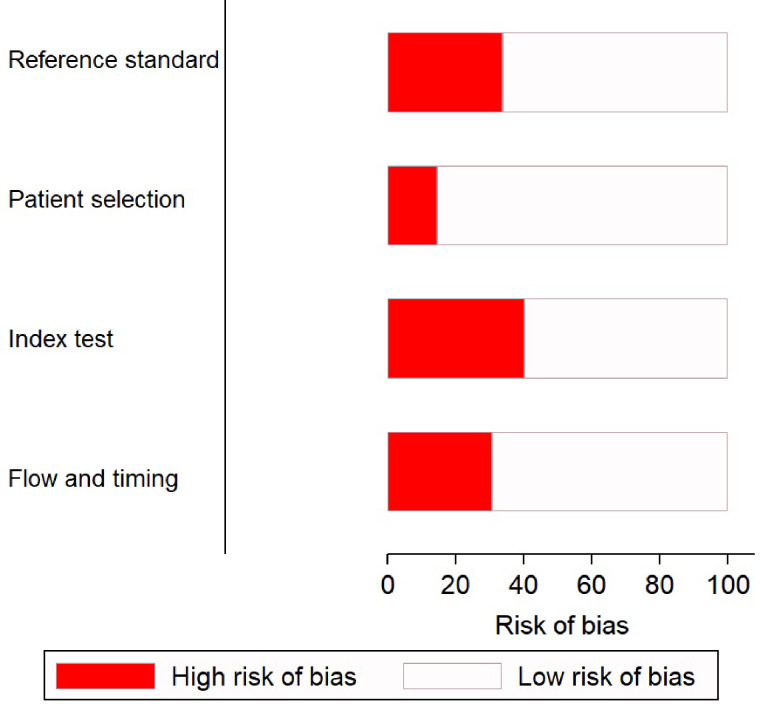
Quality assessment of the included studies (*n* = 45) using QUADAS-2 tool.

There was a low risk of patient selection bias in more than 90% of the studies (Fig. 2); 18 of 45 (see note on previous page) studies [7, 10, 11, 21–24, 30, 33–36, 38, 40, 46–50] showed a high risk of bias in conduct and interpretation of the index tests. Likewise, 16 studies [16–19, 21, 27–31, 34–36, 38, 40] had high risks of bias in conduct and interpretation of reference standards; and 14 [7, 10, 13–15, 21–27, 29, 51] had high risks of bias in patient flow and interval between index tests and reference standards.

### Diagnostic performance of semi-quantitative methods for CRBSI

**Fig. 3.**
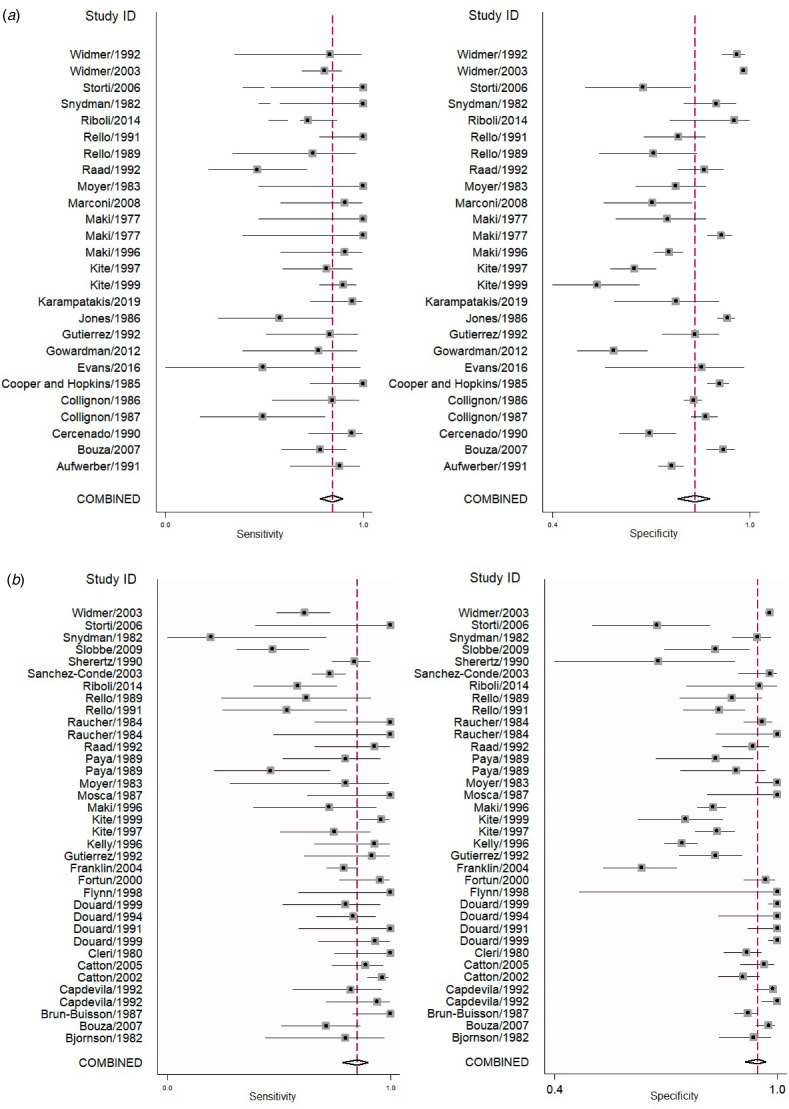
Forest plot showing pooled sensitivities and specificities. (a) For semi-quantitative methods, (b) for quantitative methods.

**Fig. 4.**
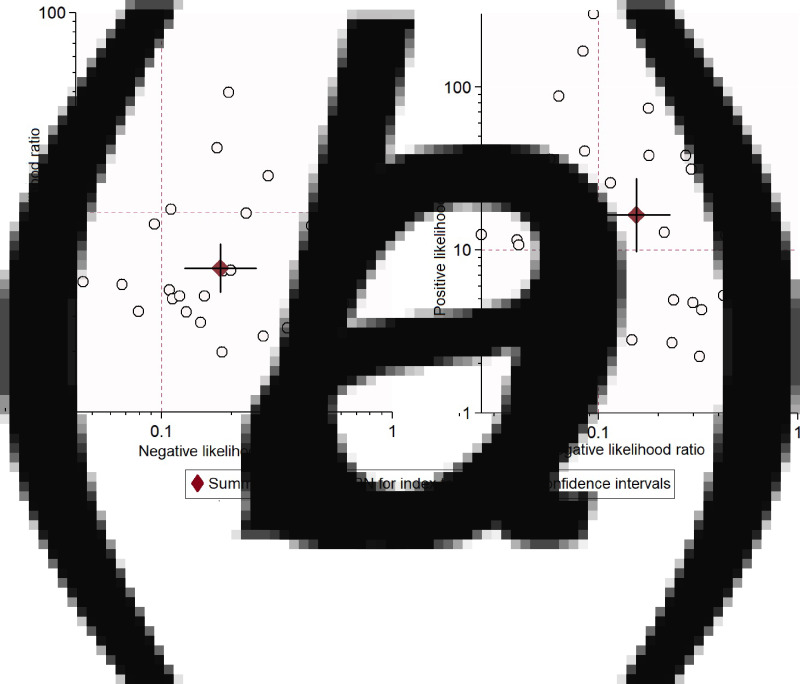
Likelihood scatter grams. (a) For semi-quantitative methods, (b) for quantitative methods. LUQ: exclusion and confirmation, LRP >10, LRN <0.1; RUQ: confirmation only, LRP >10, LRN <0.1; LLQ: exclusion only, LRP <10, LRN <0.1; RLQ: no exclusion or confirmation, LRP <10, LRN >0.1.

**Fig. 5.**
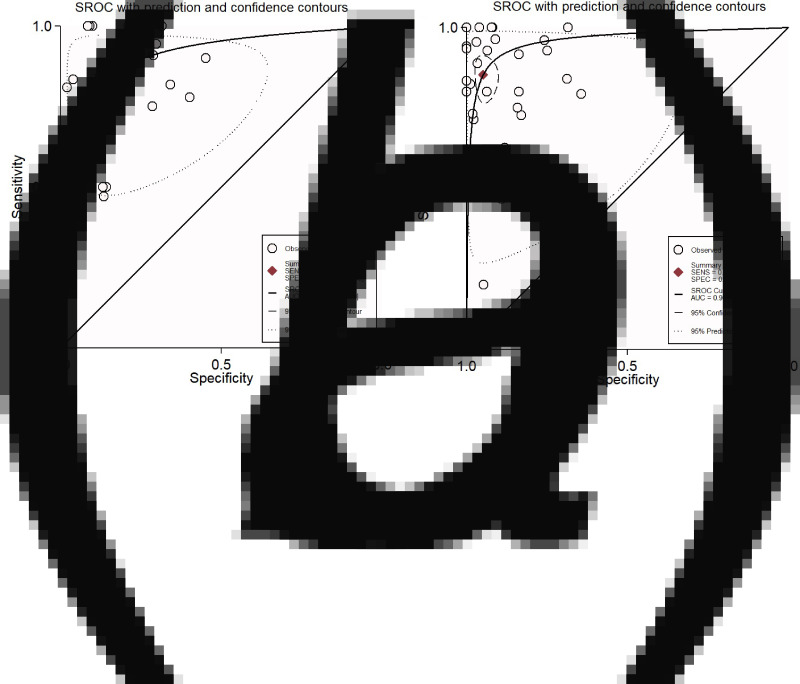
SROC curves. (a) For semi-quantitative methods in the screening of catheter-related blood stream infections. (b) For quantitative methods in the screening of catheter-related blood stream infections.

**Fig. 6.**
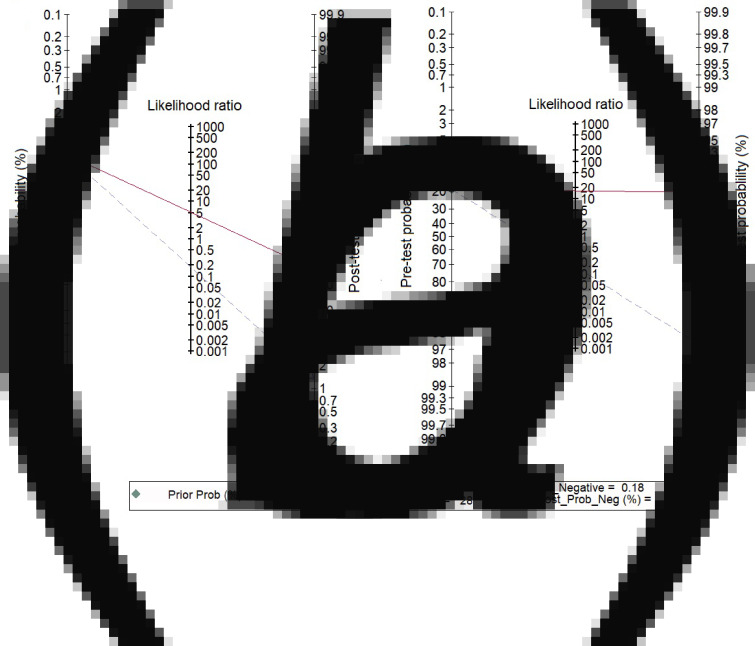
Fagan nomogram evaluating the overall values in the screening of catheter-related blood stream infections. (a) For semi-quantitative methods, (b) for quantitative methods.

We analysed 26 studies [7, 10, 12, 16, 18–20, 24, 28–31, 33–36, 38, 40, 42, 43, 46–48, 50, 52] that evaluated the diagnostic accuracy of semi-quantitative catheter segment cultures for CRBSI. Their pooled sensitivity and specificity were 85% (95% CI 79–90%) and 84% (95% CI 79–88%), respectively (Fig. 3a); the corresponding DOR was 29 (95% CI 18–47), the LRP 5.2 (95% CI 4–6.9) and the LRN 0.18 (0.13–0.26). However, as shown in Figure 4a, the LRP and LRN values fall in the right lower quadrant of the LR scattergram which indicates that the semi-quantitative methods can neither be used for confirmation nor exclusion of CRBI diagnosis. Nevertheless, the sROC curve for semi-quantitative methods gave an AUC value of 0.91 (95% CI 0.68–0.98) which was suggestive of high diagnostic performance (Fig. 5a). Likewise, Fagan's nomogram Fig. 6a) showed good clinical utility for these methods, as the post-test probability (positive = 28%; negative = 1%) differed significantly from the pre-test probability (7%).

**Fig. 7.**
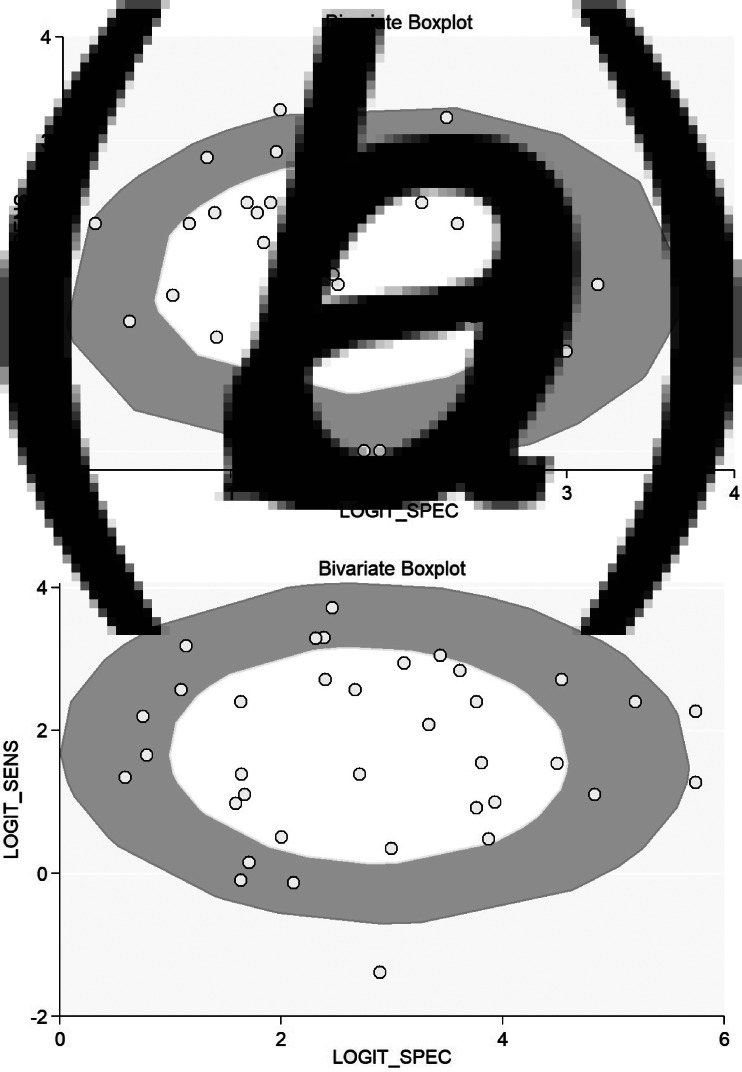
Bivariate boxplot of the sensitivities and specificities in the screening of catheter-related blood stream infections. (a) For semi-quantitative methods, (b) for quantitative methods.

**Fig. 8.**
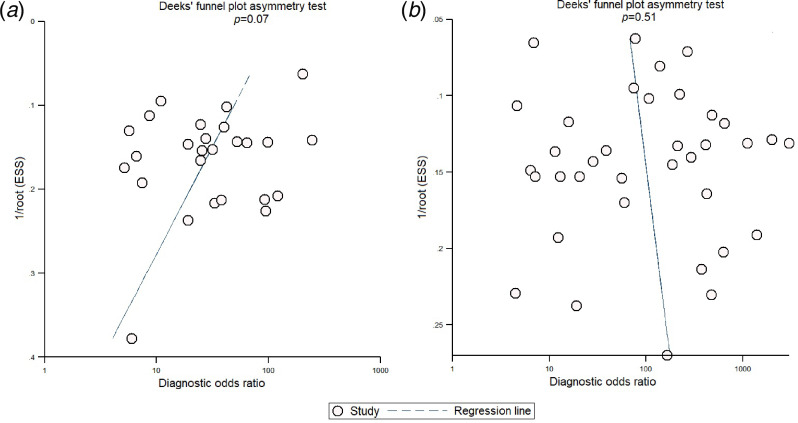
Funnel plot for assessing publication bias among studies. (a) For semi-quantitative methods, (b) for quantitative methods.

A considerable degree of heterogeneity was evident (*χ*^2^ – *P* < 0.001 – and an *I*^2^ value of 97%. The bivariate box plot (Fig. 7a) revealed two studies outside the circle implying the possibility of between-study heterogeneity. Publication bias was absent or minimal as shown by the symmetrical funnel plot (Fig. 8a) and this was confirmed by a non-significant value by Deek's test (*P* = 0.07). We explored the source of heterogeneity using subgroup analyses across regions and countries and found wide variation in the sensitivities and specificities of the semi-quantitative methods across regions; studies conducted in the Americas had maximum sensitivities (89%) and specificities (89%), while those from Australia had sensitivities as low as 71% and specificities of 80%.

### Diagnostic performance of quantitative methods for CRBSI

In total, 30 studies [7, 11–15, 17, 21–23, 25–27, 29, 32–34, 37–47, 50, 51] have evaluated the diagnostic accuracy of quantitative methods for CRBSI. The pooled sensitivity and specificity of these methods were 85% (95% CI 79–90%) and 95% (95% CI 91–97%), respectively (Fig. 3b). Their DOR was 106 (95% CI 51–221), the LRP 16.4 (95% CI 9.8–27.5) and the LRN 0.15 (0.10–0.23). LRP and LRN values were in the right upper quadrant of the LR scattergram indicating that the methods are confirmatory rather than for exclusion of the diagnosis (Fig. 4b). The AUC value was 0.96 (95% CI 0.92–0.999) and indicative of a high diagnostic performance (Fig. 5b). Fagan's nomogram (Fig. 6b) showed good clinical diagnostic utility for CRBSI as the post-test probability (positive = 79%; negative = 3%) was significantly different from the pre-test probability (19%). There was marked heterogeneity with significant *χ*^2^ (*P* < 0.001) and *I*^2^ values (99%). The bivariate box plot (Fig. 7b) revealed three studies outside the circle implying the possibility of between-study heterogeneity. Consistent with the results for semi-quantitative methods, the funnel plot was symmetrical (Fig. 8b) and the absence of publication bias was confirmed by a non-significant Deek's test (*P* = 0.51).

Our subgroup analyses based on the type of test showed that paired quantitative blood cultures had the maximum sensitivity (89%; 95% CI 74–96%; *n* = 10) and specificity (99%; 95% CI 96–100%; *n* = 10) followed by IVD-drawn quantitative blood cultures (sensitivity = 84%; 95% CI 64–94% and specificity = 94%; 95% CI 85–98%; *n* = 7), and by quantitative catheter segment cultures (sensitivity = 83%; 95% CI 74–90% and specificity = 91%; 95% CI 86–94%; *n* = 19). Sensitivity and specificity of the quantitative methods were maximal in studies from European countries.

## Discussion

Various diagnostic modalities are available for the specific diagnosis of CRBSIs and both semi-quantitative and quantitative methods are widely used for patients in tertiary care settings. However, determining the diagnostic performance of these methods is important so as to identify the best modality to be used in routine hospital care. Hence, this meta-analysis and review was designed to compare the diagnostic accuracies of both methodological approaches for such patients. We identified 45 studies with 11 232 participants fitting our eligibility criteria. Most studies were prospective in nature, had a low risk of bias with respect to the four domains in the QUADAS tool, and had been conducted in American and European countries including the USA, Brazil, Spain and France.

Our analysis suggests that semi-quantitative methods have a pooled sensitivity of 85% and a pooled specificity of 84% with a high diagnostic performance (AUC = 0.91), while quantitative methods (combining all three methods) have a similar sensitivity (85%), but higher specificity (95%) along with higher diagnostic accuracy (AUC = 0.96). Among the latter methods, paired quantitative blood cultures had the highest sensitivity (89%) and specificity (99%) followed by IVD-drawn quantitative blood cultures (sensitivity = 84% and specificity = 94%), and by quantitative catheter segment cultures (sensitivity = 83% and specificity = 91%). These diagnostic accuracy values were similar to that reported by Safdar *et al*. [8] who also concluded that paired quantitative blood cultures had the highest diagnostic performance amongst all reviewed methods.

The LR scattergram of semi-quantitative methods showed that the LRP and LRN occupied the right lower quadrant indicating that these approaches cannot be used for CRBSI exclusion or confirmation. However, our analysis suggests that quantitative methods can be used for diagnostic confirmation. The clinical values of both methodological strategies for CRBSI were high as Fagan's nomogram showed significant increases in the post-test probabilities compared to the pre-test probabilities. However, while accepting these results at face value, we must consider that different quality standards and methodologies of the studies may have influenced our summary findings. As a consequence, we evaluated the degree of heterogeneity between the studies and found this to be statistically significant. On further exploration of the source of heterogeneity via subgroup analyses, we found wide variation across regions and countries in the final pooled estimates that may have influenced the between-study variability. Nevertheless, Deek's test and funnel plot results both suggested an absence of publication bias among the studies for both semi-quantitative and quantitative methods.

Our study has some limitations. First, we found some studies to have high risks of bias, which may have influenced our final estimates. Second, the significant degree of heterogeneity limits our ability to interpret the pooled results. However, we tried to overcome this by exploring the potential source of heterogeneity among the studies. In spite of these limitations, our results provide valuable insights into the diagnostic performance of various methods for screening patients for CRBSI. Although the semi-quantitative methods had satisfactory levels of sensitivity and specificity, they did not meet the SnNout triage test criteria for sensitivity and the SpPin criteria for specificity of diagnostic tests [53]. This means that semi-quantitative methods cannot alone be used, with certainty, to confirm or exclude CRBSI in a patient. On the other hand, the quantitative methods met the SpPin criteria for specificity, indicating that they can be used with a high level of certainty to confirm CRBSI in a patient.

These findings should be considered to bring about changes in international guidelines and practices for CRBSI diagnoses. In our opinion, quantitative methods should be recommended as a first-line modality to confirm the infection in a patient. However, further studies assessing the performance of each of the quantitative methods should be carried out in different geographical regions as the evidence in low- and middle-income countries is limited. Such studies will inform the framing of guidelines and practices for patients admitted to tertiary care irrespective of the setting. Moreover, the affordability of the tests should also be considered by comparing their relative cost-effectiveness as a diagnostic modality for CRBSI.

In conclusion, both semi-quantitative and quantitative methods have high sensitivity and specificity for CRBSI screening. Quantitative methods, particularly paired quantitative culture, offer the highest sensitivity and specificity and can be used for diagnosis with a high degree of confidence. However, additional studies are warranted across all geographical regions to further inform international guidelines and practices.

## Data Availability

The datasets used and/or analysed during the current study are available from the corresponding author upon reasonable request.

## Supplementary material

For supplementary material accompanying this paper visit http://dx.doi.org/10.1017/S0950268820001673. click here to view supplementary material
