# Supplementary material for: Comparison of semi-quantitative and quantitative methods for diagnosis of catheter-related blood stream infections: a systematic review and meta-analysis of diagnostic accuracy studies
Source: Epidemiol Infect. 2020 Jul 27;148:e171. doi: 10.1017/S0950268820001673 (PMC7439295; doi:10.1017/S0950268820001673)
Supplement: Supplementary file 1 [file S0950268820001673sup001.docx]

Supplementary Table 1. Characteristics of the studies included (n=45)

| **First author and year** | **Country** | **Study design** | **Sample size** | **Type of diagnostic modality** | **Gold standard comparator** | **Study participants** | **Criteria for positivity** |
| --- | --- | --- | --- | --- | --- | --- | --- |
| Aufwerber 1991^10^ | Sweden | Retrospective | 542 | Semi-quantitative segment cultures | Qualitative catheter segment and  qualitative paired blood cultures | ICU patients | ≥15 colony forming units |
| Bjornson 1982^11^ | United States of America | Prospective | 74 | Quantitative segment blood cultures | Qualitative catheter segment and  qualitative paired blood cultures | Patients receiving total parenteral nutrition | ≥1000 colony forming units |
| Bouza 2007^12^ | Spain | Prospective | 204 | Semi-quantitative and quantitative segment blood cultures | Positive result of quantitative and/or conventional  blood cultures from a peripheral vein and semiquantitative  catheter-tip cultures positive for the same  microorganism | ICU patients | ≥15 colony forming units |
| Brun-Buisson 1987^13^ | France | Prospective | 331 | Quantitative segment blood cultures | Qualitative catheter segment and  qualitative paired blood cultures | ICU patients | ≥1000 colony forming units |
| Capdevila 1992^14^ | Spain | Prospective | 107 | IVD-drawn  quantitative  blood cultures and Paired quantitative blood cultures | Qualitative catheter segment and  qualitative paired blood cultures | ICU patients | >4:1 |
| Catton 2002^49^ | United Kingdom | Prospective | 205 | IVD-drawn  quantitative  blood cultures | Qualitative catheter segment and  qualitative paired blood cultures | Surgical patients | >100 colony forming units |
| Catton 2005^15^ | United Kingdom | Prospective | 123 | Quantitative segment  blood cultures | Qualitative catheter segment and  qualitative paired blood cultures | Surgical and ICU patients | >100 colony forming units |
| Cercenado 1990^16^ | Spain | Prospective study | 139 | Semi-quantitative segment cultures | Qualitative catheter segment and  qualitative paired blood cultures | General inpatients | >15 colony forming units |
| Cleri 1980^17^ | United States of America | Prospective study | 149 | Quantitative segment cultures | Qualitative catheter segment and  qualitative paired blood cultures | General inpatients | >1000 colony forming units |
| Collignon 1986^18^ | Australia | Prospective | 745 | Semi-quantitative segment cultures | Qualitative catheter segment and  qualitative paired blood cultures | ICU patients | ≥15 colony forming units |
| Collignon 1987^19^ | Australia | Prospective | 322 | Semi-quantitative segment cultures | Qualitative catheter segment and  qualitative paired blood cultures | Not reported | ≥15 colony forming units |
| Cooper and Hopkins 1985^20^ | United States of America | Prospective | 330 | Semi-quantitative segment cultures | Qualitative catheter segment and  qualitative paired blood cultures | ICU patients | ≥15 colony forming units |
| Douard 1991^23^ | France | Prospective | 53 | Paired quantitative blood cultures | Positive paired quantitative blood cultures | Children with  hematologic  or oncologic  illness | >5-fold |
| Douard 1994^21^ | France | Prospective | 58 | Paired quantitative blood cultures | Positive paired quantitative blood and segment blood cultures | Medical and surgical ICU patients | >3:1 |
| Douard 1999^22^ | France | Prospective | 170 | Paired and segmented quantitative blood cultures | Catheter drawn blood compared with  peripherally drawn blood | Immunocompromised patients | >4-fold |
| Evans 2016^24^ | Australia | Prospective | 16 | Semi-quantitative segmented blood cultures | Quantitative or semi-quantitative of the catheter  combined with two blood cultures | Burns patients | >15 colony forming unit |
| Flynn 1998^25^ | United States of America | Prospective | 12 | Paired quantitative blood cultures | Paired quantitative blood and segment blood cultures | Children | >5-fold |
| Fortun 2000^26^ | Spain | Prospective | 118 | Paired quantitative blood cultures | Paired quantitative blood and segment blood cultures | General inpatients | >5-fold |
| Franklin 2004^27^ | United States of America | Retrospective | 241 | IVD drawn quantitative blood cultures | Paired quantitative blood and segment blood cultures | Children with cancer | >100 Colony forming unit |
| Gowardman 2012^28^ | Australia | Prospective | 101 | Semi-quantitative segmented blood cultures | Semiquantitative  IVD tip and peripheral venous blood  cultures with the same microorganism | ICU patients | >15 Colony forming unit |
| Gutierrez 1992^29^ | Spain | Prospective | 98 | Semi-quantitative and quantitative segmented blood cultures | Quantitative and qualitative blood and segment blood cultures | General inpatients | >1000 colony forming unit |
| Jones 1986^30^ | United States of America | Prospective | 379 | Semi-quantitative segmented blood cultures | Quantitative and qualitative blood and segment blood cultures | Cancer patients | >15 Colony forming unit |
| Karampatakis 2019^31^ | Greece | Retrospective | 51 | Semi-quantitative segmented blood cultures | Quantitative and qualitative blood and segment blood cultures | ICU patients | ≥ 15 colony forming unit |
| Kelly 1996^32^ | United States of America | Retrospective | 405 | Quantitative segmented blood cultures | Quantitative and qualitative blood and segment blood cultures | General inpatients | >1000 colony forming unit |
| Kite 1997^33^ | United Kingdom | Prospective | 224 | Semi-quantitative and quantitative segment blood cultures | Qualitative blood and segment blood cultures | Surgical ICU patients | ≥1000 colony forming units |
| Kite 1999^34^ | United Kingdom | Prospective | 112 | Semi-quantitative and quantitative segment blood cultures | Qualitative blood and segment blood culture | Surgical ICU patients | ≥1000 colony forming units |
| Maki 1977^35^ | United States of America | Prospective | 50 | Semi-quantitative segment blood cultures | Qualitative blood and segment blood cultures | Burns patients | ≥15 colony forming units |
| Maki 1977^6^ | United States of America | Prospective | 250 | Semi-quantitative segment blood cultures | Qualitative blood and segment blood cultures | General inpatients | ≥15 colony forming units |
| Maki 1996^50^ | United States of America | Prospective | 400 | Semi-quantitative and quantitative segment blood cultures | Culture isolates of  hub, infusion, or  catheter segment  and bloodstream  infections by DNA  subtyping | General inpatients | ≥15 colony forming units |
| Marconi 2008^36^ | Brazil | Prospective | 63 | Semi-quantitative segment blood cultures | Same microorganism (species and profile of susceptibility to agents) isolated from both catheter tips and peripheral blood cultures | Newborns | ≥100 colony forming units |
| Mosca 1987^37^ | United States of America | Prospective | 26 | Paired quantitative segment blood cultures | Clinical follow-up | General inpatients | >5-fold |
| Moyer 1983^38^ | United States of America | Prospective | 73 | Semi-quantitative segment and IVD drawn quantitative blood cultures | Qualitative blood and segment blood cultures | Patients receiving total parenteral nutrition and patients with burns | ≥25 colony forming units |
| Paya 1989^39^ | United States of America | Prospective | 52 | IVD drawn and paired quantitative blood cultures | Qualitative blood and segment blood cultures | Surgical patients | Any growth |
| Raad 1992^40^ | United States of America | Prospective | 128 | Semi-quantitative segment and quantitative segment blood cultures | Qualitative blood and segment blood cultures | General inpatients | ≥1000 colony forming units |
| Raucher 1984^41^ | United States of America | Prospective | 137 | IVD drawn and paired quantitative blood cultures | Qualitative blood and segment blood cultures | Children | >5:1 |
| Rello 1989^42^ | Spain | Prospective | 50 | Semi-quantitative segment and quantitative segment blood cultures | Qualitative blood and segment blood cultures | End stage renal disease patients | ≥1000 colony forming units |
| Rello 1991^43^ | Spain | Prospective | 98 | Semi-quantitative segment and quantitative segment blood cultures | Qualitative blood and segment blood cultures | General inpatients | ≥1000 colony forming units |
| Riboli 2014^7^ | Brazil | Retrospective | 50 | Semi-quantitative segment and quantitative segment blood cultures | Qualitative blood and segment blood cultures | Newborns | ≥1000 colony forming units |
| Sanchez-Conde 2003^51^ | United States of America | Prospective | 197 | Paired quantitative blood cultures | Qualitative blood and segment blood cultures | Adults | ≥5-fold |
| Sherertz 1990^44^ | United States of America | Retrospective | 97 | Quantitative blood cultures | Qualitative blood and segment blood cultures | ICU patients | ≥1000 colony forming units |
| Slobbe 2009^45^ | Netherlands | Prospective | 89 | Quantitative segment blood cultures | Qualitative blood and segment blood cultures | Adults with hemotological disease | ≥15 colony forming units |
| Snydman 1982^46^ | United States of America | Prospective | 100 | Semi-quantitative segment and IVD-drawn quantitative blood cultures | Qualitative blood and segment blood cultures | Patients receiving total parenteral nutrition | Any growth |
| Storti 2006^47^ | Brazil | Prospective | 42 | Semi-quantitative segment and quantitative segment blood cultures | Qualitative blood and segment blood cultures | ICU patients | ≥15 colony forming units |
| Widmer 1992^48^ | United States of America | Prospective | 157 | Semi-quantitative segment drawn blood cultures | Qualitative blood and segment blood cultures | ICU patients | ≥15 colony forming units |
| Widmer 1992^52^ | United States of America | Prospective | 1000 | Semi-quantitative segment drawn blood cultures | Qualitative blood and segment blood cultures | Not reported | ≥15 colony forming units |
